# Assessing Risk Factors for Migraine: Differences in Gender Transmission

**DOI:** 10.1371/journal.pone.0050626

**Published:** 2012-11-21

**Authors:** Carolina Lemos, Isabel Alonso, José Barros, Jorge Sequeiros, José Pereira-Monteiro, Denisa Mendonça, Alda Sousa

**Affiliations:** 1 UnIGENe, Instituto Biologia Molecular Celular, Universidade do Porto, Porto, Portugal; 2 Instituto Ciências Biomédicas Abel Salazar, Universidade do Porto, Porto, Portugal; 3 Serviço de Neurologia, Centro Hospitalar do Porto, Hospital de Santo António, Porto, Portugal; 4 Instituto de Saúde Pública, Universidade do Porto, Porto, Portugal; “Mario Negri” Institute for Pharmacological Research, Italy

## Abstract

**Aim:**

Our aim was to assess which specific factors are contributing to an increased risk of migraine in a group of 131 Portuguese families.

**Methods:**

We studied 319 first-degree relatives, using a multilevel approach to account for the dependency among members from the same family. We included in the model relative’s gender, the proband’s gender and age-at-onset, to evaluate if any of these variables were associated with relative’s affection status. We also included in the model proband’s migraine subtype. We further assessed female and male transmissions within the proband nuclear family.

**Results:**

Relatives’ gender was found to be a risk factor for migraine (Odds Ratio = 2.86; 95% CI = 1.75–4.67), with females at a higher risk. When splitting probands according to their migraine subtype, we found that none of the variables studied contributed to relatives of MA-probands affection-status. Our results also show a significant difference between proband’s transmission and the gender of the parents and offspring.

**Conclusions:**

With this study, we showed that gender is truly a risk factor for migraine and that a gender-biased transmission is also observed. This reinforce the importance of identifying genes associated with migraine that are modulated by genes located in the sex chromosomes and the study of mitochondrial DNA or X-chromosome and hormonal-related effects associated with migraine susceptibility.

## Introduction

Complex diseases have a high impact on human health and a high population incidence [1]. Migraine, a highly prevalent disease, is one example among many others [2]. Migraine with (MA) and without aura (MO) are the most common forms of this disease. It is a disabling disease leading to a diminished quality of life in both migraineurs and their relatives [3].

The consistent finding of an increased risk for relatives of migraineurs suggests that genetic factors may be implicated in the most common forms of the disease [2]. We also found a substantial familial risk of migraine for first-degree relatives in a sample of Portuguese migraineurs, which has led us to conclude that migraine could be strongly due to genetic factors [4].

In a previous study, a lower age at onset in probands was found to be a predictor of migraine familial aggregation [5]. Lifetime prevalence of migraine is increased in females compared to males, with a female:male ratio ranging from 2∶1 to 4∶1 in several populations [6], [7], [8], [9]. Several hypotheses have been raised for this female predominance such as neurobiological factors, increased sensitivity to environmental stressors or a greater genetic loading for migraine [10], [11].

Our aim now was to assess which specific factors from our migraine families are contributing to the increased risk for this disorder, taking into account that observations within the same family are not independent.

## Materials and Methods

### Data Collection

Sample collection was described in a previous study of our group [4]. A diagnostic interview of probands and first-degree relatives (parents, sibs and offspring) was performed using the same structured questionnaire based on the diagnostic criteria of the “International Headache Society” (IHS). To avoid a selection bias towards affected relatives, family members were contacted regardless of the information provided by the proband about their affection status. The first edition of the IHS criteria (ICHD-I) [12] was used before 2004; when revising the diagnosis using the second edition (ICHD-II) [13] no differences were found in patients’ diagnosis (data not shown).

### Ethics Statement

Participants gave their written informed consent and the Ethics Committee of CHP,HSA approved the project.

### Data Analysis

Probands were classified according to their migraine subtype. Proband’s age at onset was dichotomized (<16, 16+ years) according to the criterion of Stewart et al [5]. Relatives were divided into three groups according to their age-at-observation (<30, 30–59, 60+ years) in order to obtain a homogeneous distribution of the individuals in each group, and were also stratified by gender, since migraine is an age and gender-dependent trait.

We also assessed female and male transmissions within the proband nuclear family. We excluded from this analysis bilinear transmissions, i.e. transmissions where both the mother and the father were affected.

### Statistical Analysis

A multilevel generalised linear analysis using a logit model for a binary variable of outcome (affected/non-affected) was conducted to account for the non-independency among members from the same family, with relatives nested within the families. The multilevel modelling has been described as an advantageous tool for modelling data with a hierarchical structure [14].

We included in the model relative’s gender, proband’s gender and age-at-onset as independent variables, to evaluate if any of these variables were associated with relative’s affection status in our families. We also included in the model proband’s migraine subtype and relative’s age-at-observation. Independent variables were adjusted by analyzing their possible effects on the outcome (affection status) altogether in the model. The multilevel generalised linear model was fit using MLwiN 1.10 software.

Categorical data were compared by a chi-square test, using SPSS version 16.0 for Windows. A 5% significance level was used in all analyses.

## Results

### Demographic Data

#### Probands

A total of 131 probands were enrolled in this study (104 women and 27 men, mean age ± SD, 34.4±12.7 years). Regarding age-at-onset, 60 probands had an age-at-onset below 16 years old, while 71 showed migraine after that age. When classifying probands according to their migraine subtype, 85 probands had MO while 46 had MA.

#### Relatives

Interviews were conducted with 182 first-degree relatives of MO-probands (114 women and 68 men) and 137 first-degree relatives of MA-probands (79 women and 58 men).

In MO-probands group, 116 were affected while 66 were non-migraineurs. In the MA-probands-group, 81 family members were affected while 56 were not. Age of relatives was included in the model to adjust for this variable, since migraine is an age-dependent trait. From the total of first-degree relatives, 112 were parents, 139 were siblings whereas 68 were offspring.

### Risk Factors

After adjusting for the remaining variables, relatives’ gender was found to be a risk factor for migraine (OR = 2.86; 95% CI = 1.75–4.67) (Table 1), with females first-degree relatives at higher risk than males. Variables related with proband’s, gender and age-at-onset were not risk factors for migraine (p>0.05). When introducing in the model proband’s migraine subtype this variable also did not influence relative’s affection status (p>0.05).

**Table 1:** Risk factors of migraine: results from the multilevel model, adjusted for relative’s age at observation (Odds ratio, 95% CI).

Variables	Migraine Probands	MO Probands	MA Probands
Proband’s gender	0.79 (0.39–1.62)	0.61 (0.22–1.74)	1.16 (0.41–3.31)
Proband’s age at onset	0.75 (0.42–1.33)	0.50 (0.23–1.10)	1.21 (0.48–3.09)
Relative’s gender	2.86 (1.75–4.67)	4.15 (2.06–8.34)	1.86 (0.90–3.83)
Proband’s migraine subtype	0.78 (0.45–1.36)	–	–

Relative’s gender, proband’s gender and age-at-onset were included in the model as possible predictors of relatives’ affection status. Proband’s migraine subtype was also included as a predictor in an additional model.

When splitting probands according to their migraine subtype, we found that none of the variables studied contributed to relatives of MA-probands affection-status. Conversely, gender of relatives of MO-probands was associated with their affection status (OR = 4.15; 95% CI = 2.06–8.34) (Table 1).

### Maternal and Paternal Transmissions

Relatives’ gender was found to be a risk factor for migraine, with females being at a 3-fold higher risk than males. Therefore we hypothesized that gender ratio in migraine families could be due to a biased transmission. In order to explore this hypothesis we analysed female and male transmissions within the proband’s nuclear family. Our result showed a significant difference between proband’s transmission and the gender of the offspring (Table 2) as we found that daughters are more affected than expected (X^2^_(1)_ = 6.91, p = 0.009).

**Table 2:** Distribution of affected and non-affected offspring and parents according gender.

Offspring	Affected (%)	Non-affected (%)	Total	Parents	Affected (%)	Non-affected (%)	Total
Daughters	27 (75)	9 (25)	36	Mothers	60 (78)	17 (22)	77
Sons	14 (44)	18 (56)	32	Fathers	11 (31)	24 (69)	35

We also found that mothers of probands are more affected than expected when compared to proband’s fathers (X^2^_(1)_ = 22.41, p<0.001) (Table 2).

We also compared the ratio of affected fathers, mothers and siblings. We found a higher ratio of affected mothers (78%) and siblings (61%) than affected fathers (30%), with mothers being affected two to three times more than fathers.

## Discussion

Familial aggregation is well established for common migraine, nevertheless, several familial factors may be contributing for these increased risk. Our aim was to search for migraine predictors in a sample of Portuguese migraine families.

In a previous study, a lower age at onset in probands was associated with relative’s affection status [5]; in contrast, in our study, the proportion of affected relatives was independent of proband’s age at onset. After adjusting for the remaining variables, gender was found to be the only risk factor for migraine, while proband’s age at onset and relative’s age at contact were not.

Our findings showed that, as expected, females had a higher risk of migraine than men. Our next aim was then to assess if there was a gender-biased transmission. We found that mothers of probands were more frequently affected than expected. This biased transmission could be explained by a maternally inherited factor such as mitochondrial DNA (mtDNA) [15], [16]. We also found that the ratio of affected probands’ fathers is lower than the ratio of affected mothers and siblings which is an evidence in favour of a maternally inherited factor, according to Boles et al [15]. Although mtDNA may not explain by itself the gender differences found, since migraine is a complex disease with several genetic factors involved, variants in mtDNA or in nuclear genes affecting mitochondrial mechanisms could influence migraine susceptibility. Also it has been suggested that an impairment of mitochondrial metabolism could lower the threshold for migraine attacks [17].

As suggested by Boles et al, we assessed if offspring of probands would be differentially affected and in fact we found that daughters of probands are more affected than expected. Other authors found evidence of a migraine susceptibility locus on chromosome × which could explain the daughters’ increased frequency [18], [19], [20], [21]. Another hypothesis is a sex-conditioned genetic model with sex chromosomes influencing the expression of genes in autosomes, which may explain the different prevalence in female and male family members [22]. This was already observed in other human traits as genetic baldness, where both males and females are affected but with different ratios. Furthermore, specific inter-chromosome interactions have been observed in some neurological diseases where the mutation responsible for the disease located in one chromosome may modify the expression of genes located in another chromosome [22]. Additionally, in our sample we found an enrichment of the G allele of rs6951030 in the *STX1A* gene for female migraineurs only, which reinforces a gender-specific susceptibility in migraine [23].

Migraine presents different gender thresholds, with males having a higher threshold [11], [24]. Female steroids play an important role in migraine pathophysiology and can also explain the differential gender ratio found for this disorder since they are involved in mechanisms related in migraine pathophysiology, such as in neuronal excitability, in the synthesis and release of nitric oxide (NO) and neuropeptides such as calcitonin-gene related peptide (CGRP). Also, the serotonergic, adrenergic and GABA-ergic systems are also modulated by female steroids. Furthermore, some variants in female hormones receptors, such as estrogens and progesterone receptors have been found to be associated with migraine susceptibility [25], [26], [27].

Our findings regarding risk factors for migraine subtypes lead us also to hypothesize that in our sample, gender-risk factors may not be associated with MA susceptibility, while in MO, hormonal events could influence the risk of having migraine, showing a conjunction of environmental and genetic factors, as suggested previously by Russell et al [28]. Although our sample size can be a limitation of our study, we had some special concerns in the design of the study and in the sample ascertainment. In this study we have taken into account intrafamilial correlations, by using a statistical analysis which corrects for this fact. Male probands are in small number due to a lower frequency of migraine in males and although the number of first-degree relatives of male probands is smaller than female probands, this was not due to an ascertainment bias, since probands were selected regardless of their family history. Furthermore, as described previously [4], some first-degree relatives were contacted by telephone, a method that has been described as a valid instrument [9], [29] and that circumvent the possibility of an unbalanced ratio of affected females coming to interviews. Hence, by telephone, both females and males were contacted, avoiding a female preponderance bias.

With this study, we reinforce the importance of identifying genes associated with migraine that are modulated by genes located in the sex chromosomes and the study of mtDNA or X-chromosome and hormonal-related effects. These studies will be crucial to bring some light into migraine’s susceptibility and in particular, gender-specific liability.
